# Microbiome of the Southwestern Atlantic invasive scleractinian coral, *Tubastraea tagusensis*

**DOI:** 10.1186/s42523-020-00047-3

**Published:** 2020-08-11

**Authors:** Aline Aparecida Zanotti, Gustavo Bueno Gregoracci, Katia Cristina Cruz Capel, Marcelo Visentini Kitahara

**Affiliations:** 1 Programa de Pós Graduação em Sistemas Costeiros e Oceânicos (PGSISCO), Universidade Federal do Paraná (UFPR), Pontal do Paraná, Brazil; 2 Centro de Biologia Marinha (CEBIMar), Universidade de São Paulo (USP), São Sebastião, Brazil; 3 Departamento de Ciências do Mar (DCMar), Universidade Federal de São Paulo (UNIFESP), Santos, Brazil

**Keywords:** Coral, Bacteria, Core microbiome, Amplicon metagenomics

## Abstract

**Background:**

Commonly known as sun-coral, *Tubastraea tagusensis* is an azooxanthellate scleractinian coral that successfully invaded the Southwestern Atlantic causing significant seascape changes. Today it is reported to over 3500 km along the Brazilian coast, with several rocky shores displaying high substrate coverage. Apart from its singular invasiveness capacity, the documentation and, therefore, understanding of the role of symbiotic microorganisms in the sun-coral invasion is still scarce. However, in general, the broad and constant relationship between corals and microorganisms led to the development of co-evolution hypotheses. As such, it has been shown that the microbial community responds to environmental factors, adjustment of the holobiont, adapting its microbiome, and improving the hosts’ fitness in a short space of time. Here we describe the microbial community (i.e. Bacteria) associated with sun-coral larvae and adult colonies from a locality displaying a high invasion development.

**Results:**

The usage of high throughput sequencing indicates a great diversity of Bacteria associated with *T. tagusensis*, with *Cyanobacteria*, *Proteobacteria*, *Bacteroidetes*, *Actinobacteria*, *Planctomycetes,* and *Firmicutes* corresponding to the majority of the microbiome in all samples. However, *T. tagusensis’* microbial core consists of only eight genera for colonies, and, within them, three are also present in the sequenced larvae. Overall, the microbiome from colonies sampled at different depths did not show significant differences. The microbiome of the larvae suggests a partial vertical transfer of the microbial core in this species.

**Conclusion:**

Although diverse, the microbiome core of adult *Tubastraea tagusensis* is composed of only eight genera, of which three are transferred from the mother colony to their larvae. The remaining bacteria genera are acquired from the seawater, indicating that they might play a role in the host fitness and, therefore, facilitate the sun-coral invasion in the Southwestern Atlantic.

## Introduction

During the last decades, rocky shore communities on the Brazilian coast have been severely impacted by the invasive scleractinian corals *Tubastraea tagusensis,* and *T. coccinea* [1–4]. The genus *Tubastraea* was first recorded in the Southwestern Atlantic on oil platforms in the late 1980’s [5] and, today its distribution extends for more than 3500 km of the Brazilian coast on natural and artificial substrates [1, 6–11]. At some localities, these invasive corals nearly saturate the substrate [12], outcompeting native and endemic species [13], and triggering seascape changes [3, 14].

The successful invasion of both species has been related to their fast growth rate, early reproductive age, the year-round release of long-lived clonal larvae, high regenerative capacity, and lack of native competitors/predators [13, 15, 16]. Furthermore, unlike most of their natural distributional range, the majority of the Brazilian invaded localities are mesotrophic, which ultimately provides a more stable food supply needed to maintain azooxanthellate species (i.e. those lacking the symbiosis with photosynthetic dinoflagellates).

Less understood, however, is the role of the microbial community in *Tubastraea* spp. fitness. Overall, scleractinian corals are believed to harbor one of the most complex communities of symbiont microorganisms including bacteria, archaea, fungi, and viruses, which collectively are known as the ‘microbiome’ [17]. Purported to enhance the hosts’ fitness and functioning [17], the coral microbiome is the central part of a widespread hypothesis of co-evolutive processes known as holobiont [18, 19]. Taking into account factors such as location, environmental changes, depth, bleaching, and diseases, the microbiome of more than 30 coral species has already been studied [20–26]. Focusing on Bacteria, it is suggested that *Proteobacteria*, *Bacteroidetes*, *Firmicutes*, *Actinobacteria*, and *Cyanobacteria* are predominant [20] and that their diversity and abundance are linked to biotic and abiotic factors such as seasonality, anthropic impacts, temperature changes, diseases, etc. [20]. Among the explanations for the occurrence of these predominant bacterial phyla in the microbiome, it is put forward that there are species-specific operational taxonomic units (OTUs) resulting from co-evolutionary processes (host and microbiota) and vertical transfer of microbiota [27, 28] that remain associated to the host regardless of environmental condition. Commonly referred to as the “microbial core”, this microbiota is considered crucial to the host health [20]. On the other hand, the fluctuating portion of the microbiota is the result of a manifold of environmental pressures acting on the host [29].

Overall, the symbiotic relationship between eukaryotes and bacteria are not only ubiquitous [27, 30, 31], but a key question within evolutionary studies [32], where the former does not figure as autonomous entities, but rather as holobionts [27, 33, 34]. Such relationships reflect on fitness and adaptation [35], enabling a faster adjustment of the holobiont to environmental changes by adapting its microbiome, ultimately influencing evolution if the latter is transmitted between generations [19, 36].

Although studies focused on understanding the microbiome of corals have increased in the past decade [20, 22, 37–39], azooxanthellate scleractinians remain understudied, with only six species analyzed to date: *Madrepora oculata* [40]*, Lophelia pertusa* [40, 41]*, Tubastraea coccinea* [38, 42], *Dendrophyllia* sp*.*, *Eguchipsammia fistula*, and *Rhizotrochus typus* [43]*.* Using a metagenomic approach, the present study describes the microbial community of *T. tagusensis* from an invaded locality in the Southwestern Atlantic, evaluates how depth influences its microbiome, and investigates the hypothesis of vertical transmission of the bacterial core between coral generations (i.e. adult colonies to larvae).

## Results

### The microbiome of *T. tagusensis* adults

Despite several attempts, the PCR step was successful for only six colonies of the first sampling (October 9th, 2017) representing two colonies per depth strata (3, 6, and 8 m). The average number of reads per colony was 11,033, ranging from 6190 to 16,864. A total of 32,070 eukaryote reads were identified and removed from subsequent analyses, along with all sequences identified as chloroplasts, mitochondria, and unknown. From the remaining sequences, an average of 1188 bacterial reads per coral sample were identified (Table 1) and classified to the genus level (except for those indicated in Additional file 1: Table S1).

**Table 1:** Richness indices (RI), Shannon H diversity (SHD), Effective number of species (Neff Shannon/exp. H), Simpson index (D), Effective number of species (Neff/D2/inverse Simpson) based on the total reads found in each sample

	*Sample*	*Total reads*	*RI*	*SHD*	*Neff Shannon/exp H*	*D*	*Neff/D2/Inverse Simpson*
*3 m*	A	1110	113	2.73	15.27	1.77E-01	5.65
	B	1939	70	1.07	3.05	6.04E-01	1.65
*6 m*	C	793	73	1.89	6.63	4.10E-01	2.44
	D	676	78	2.69	14.69	1.55E-01	6.46
*8 m*	E	865	93	2.31	10.03	2.48E-01	4.04
	F	1734	71	1.44	4.21	3.88E-01	2.58
*larvae*	G	11,439	94	1.07	2.91	6.32E-01	1.58

Although the total number of bacterial reads from the larval pool was significantly higher (11,459) than that from adult colonies (677–1943), on average, they had similar richness index (RI). However, the number of effective species based on Simpson and Shannon indexes was smaller for the larvae, indicating the dominance of only a few bacterial groups. Among adults, one colony sampled at 3 m and one from 6 m showed the highest effective number of species (ENS) (Shannon Neff = 15.27 and 14.69, Simpson Neff = 5.65 and 6.46, respectively), while the other sampled at 3 m had the lowest ENS values (Shannon Neff = 3.05, Simpson Neff = 1.05). The average ENS detected did not reflect a depth gradient nor interconnection between samples of the same depth (Table 1).

**Fig. 1:**
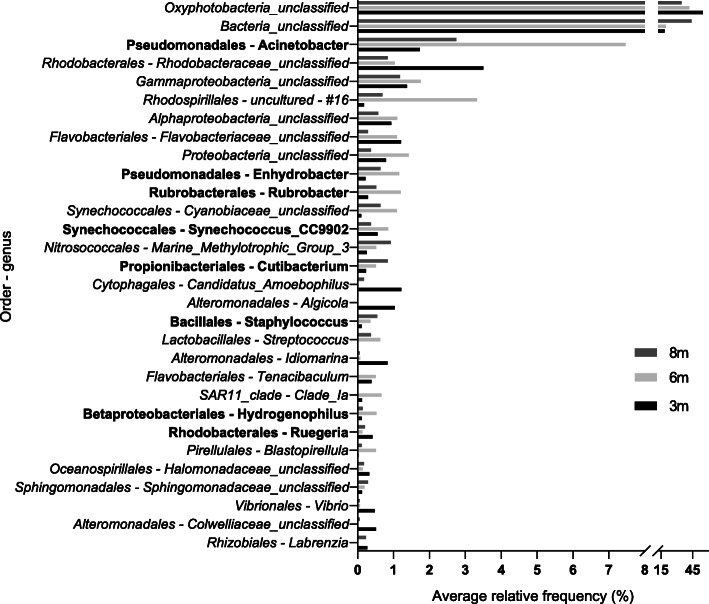
Average relative frequency of the 30 most abundant associated with the colonies of *Tubastraea tagusensis* from 3, 6, and 8 m depth. OTU’s in bold represent the core microbiome

**Fig. 2:**
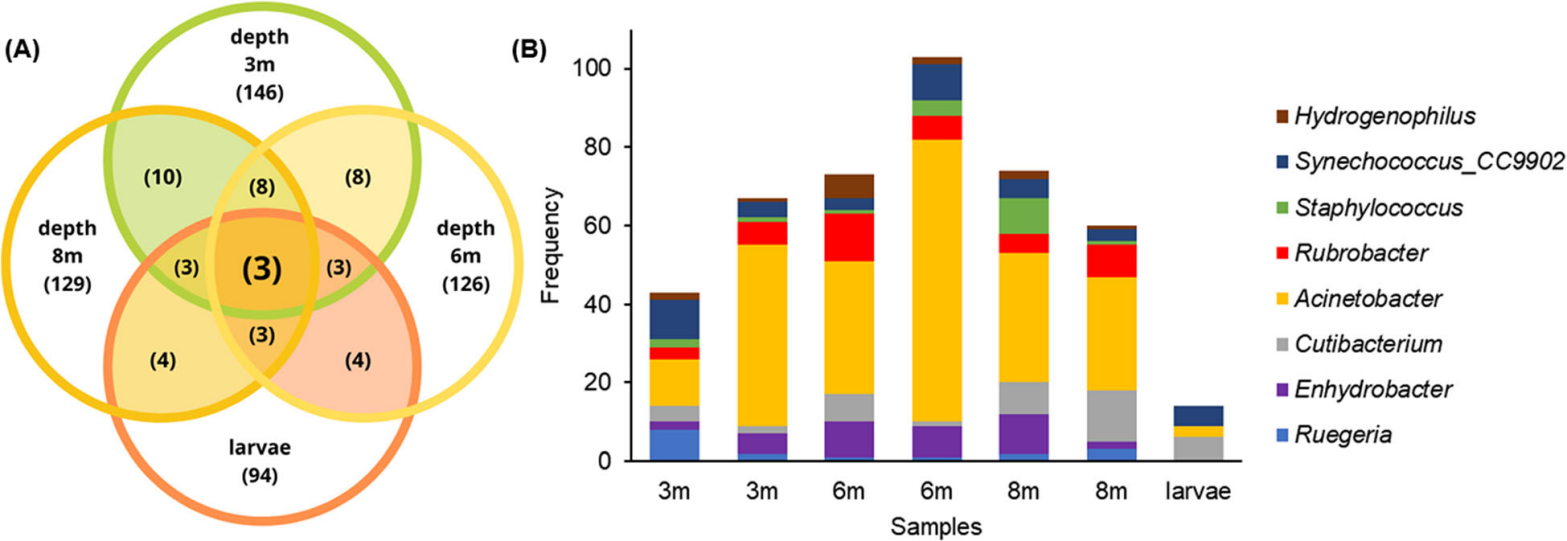
Bacterial genera shared by different specimens of *Tubastraea tagusensis* (i.e. colonies sampled at different depths and larvae). **a** Venn diagram indicating the number of OTUs from *T. tagusensis* adult colonies per sampling depth and larvae, and the number of genera common to all analyzed samples. **b** Frequency of each of the eight genera in each sample that make up the microbial core of *T. tagusensis*, including adults and larvae

A total of 15 phyla of Bacteria were identified, among which six were present in all adult samples (Table 2). Within these phyla, 236 OTUs were retrieved (Additional file 2: Figure S1 [44]) and corresponded to 167 bacteria identified to genus level, 15 as uncultured, and 81 classified only to a higher level (family, order, class, and phylum), implying uncultured microorganisms. Among all classifications, 30 OTUs are responsible for the largest portion of the microbiome (Fig. 1). Sixteen OTUs (representing ~ 84% of the whole microbial abundance) were recovered in all analyzed colonies, of which eight were classified to the genus rank and correspond to the “microbial core” for the Southwestern Atlantic invasive *T. tagusensis* (Fig. 2b).

**Table 2:** Relative frequency of the most common bacterial phyla from samples in relation to sampling depth

Domain	Phylum	Relative frequency (%)	Relative frequency (%)	Relative frequency (%)
		3 m	6 m	8 m
Bacteria	*Cyanobacteria*	55.92	44.72	36.14
	*Proteobacteria*	19.28	25.28	13.80
	*Bacteroidetes*	4.57	3.21	1.60
	*Actinobacteria*	0.66	3.54	1.98
	*Planctomycetes*	0.39	1.05	0.60
	*Firmicutes*	0.24	1.51	1.66
	Total	81.08	79.33	55.80

The eight bacterial genera that compose the microbial core of *T. tagusensis* make up only a small portion of the microbiome associated with this coral (3.66, 12.22, and 6% of the whole microbiome from colonies sampled at 3, 6, and 8 m). Overall, no significant differences in the microbial community from colonies sampled at different depths (3, 6, and 8 m - ANOVA) or depth groups (3 and 6 m, 3 and 8 m, and 6 and 8 m, Whites’ non-parametric t-test) were observed. Nevertheless, although *T. tagusensis* from different depths host a somewhat similar number of OTUs, their frequencies were variable (Fig. 2).

### The microbiome of *T. tagusensis* larvae

Sequencing of the larval pool resulted in 20,921 taxonomic classifications, of which 9465 represented eukaryotes, chloroplasts, mitochondria, and unknown sequences, and the remaining represented bacteria. It was not possible to amplify the mother colony of these larvae with the protocol used. Totaling ten phyla, the diversity of microbes found in the larvae is similar to that observed from adult colonies, albeit at different proportions. Most of the larval microbiome is composed of *Bacteroidetes* (88.43%), followed by unclassified Bacteria (5.71%), *Proteobacteria* (3.27%), and *Cyanobacteria* (1.93%). Among the phyla, 89 OTUs were retrieved, of which 55 genera and one uncultured bacteria were identified, indicating that the vast majority (~ 85%) of the larval symbiont community is composed of *Flavobacteriales* and unclassified *Flavobacteriaceae* (79.33%), unclassified Bacteria (5.74%), *Spongiiferula* (4.04%), and unclassified *Bacteroidia* (2.41%).

*Tubastraea tagusensis* larvae share 50 OTUs with the adult colonies, totaling 97.5 and 86.7% of the microbial community from larvae and adults, respectively. Larvae also hosted three out of eight genera that compose the microbial core from the adult colonies, differing by their relative frequency (which was lower in the larvae) and by the absence of *Ruegeria* (order *Rhodobacterales*), *Enhydrobacter* (order *Pseudomonadales*), *Rubrobacter* (order *Rubrobacterales*), *Staphylococcus* (order *Bacillales*), and *Hydrogenophilus* (order *Betaproteobacteriales*). Therefore, the microbial core is variable according to the developmental stage of the species (i.e. colonies and larvae) (Fig. 2).

## Discussion

The microbial community associated with Southwestern Atlantic invasive *T. tagusensis* is quite diverse. Among the 15 bacterial phyla (264 OTUs), eight compose its microbial core when adults, and three compose the larval microbial core. Such a difference in the composition of the microbial core of larvae and adults has previously been observed in other scleractinians, such as *Acropora digitifera* [21]. A similar bacterial diversity with an analogous composition has been observed for other shallow-water scleractinians such as *Mussismilia braziliensis*, *M. hispida*, *Madracis decactis*, *Tubastraea coccinea*, *Porites astreoides,* and *Acropora millepora* [38, 45, 46], and also from the zoantharian *Palythoa caribaeorum* [38]. Furthermore, Blackall et al., [17] also point to a similar frequency and abundance of the same phyla in metagenomic studies of other coral species. One of the most abundant bacterial phylum associated with invasive *T. tagusensis* (i.e. *Proteobacteria*), is not only the most abundant in the marine environment [47] but has also been reported associated with Brazilian corals, such as *Siderastrea stellata* [48] and *Mussismilia hispida* [38, 49], and *Porites astreoides* [50], in addition to *P. lutea* [51], *Pocillopora damicornis* [52]*,* and *Platygyra carnosa* [53] from other Atlantic localities or ocean basins.

Microbiome analyses of water samples from Ilha dos Búzios have contained the same main bacterial phyla as that observed in association with *T. tagusensis* [38]. Such similarity between the seawater microbiota and that associated with corals might indicate: 1) a strategy of obtaining the microbiota, which in this case would be represented by horizontal acquisition (coral microbiome would be obtained from the environment and the association would improve host’s fitness), or 2) an “ancient” relationship coupled with co-evolution of the host and their microbiome. However, additional studies are necessary to better understand the relationship between the water and coral *T. tagusensis* microbiomes in a lower taxonomical level. Previous studies have shown that the stability of a microbiome community is host-specific and, under different environmental conditions, some hosts maintain the microbial community (e.g. *Pocillopora verrucosa*) while others seem to adapt (e.g. *Acropora hemprichii*) [24]. Although the inclusion of samples from other localities/environmental conditions and the native range of *T. tagusensis* is necessary to test the hypothesis of horizontal transmission, the ability to adapt their microbiome to local environmental conditions would be another tool improving its invasive capabilities.

Within the diversity observed herein, eight OTUs compose on overage 7% of the microbiome in all specimens examined. Therefore, these were considered so far as the microbial core of *T. tagusensis*. The presence of a microbial core indicates that some genera of bacteria could be crucial to the host [20]. Interestingly, even though *Ralstonia* (*Burkholderiaceae*) and *Propionibacterium* (*Propionibacteriaceae*) have also been reported as core microbiome members in zooxanthellate coral species [37], a different genus belonging to the same families was identified in *T. tagusensis* (i.e. *Cutibacterium*, order *Propionibacteriaceae*). Representatives from this family have also been considered core microbiome of other species of azooxanthellate scleractinians, such as *Dendrophyllia* sp., *Eguchipsammia fistula* [43], and *Desmophyllum pertusum* [22], as well as in the octocorallians *Paramuricea placomus* [54], *Anthothela grandiflora*, *Anthothela* sp., *Lateothela grandiflora*, *Paramuricea placomus*, *Primnoa pacifica*, and *P. resedaeformis* [22]. The persistence of a family in the microbial core of these species may be an indication of a long-standing co-evolution between coral lineages and their microbiome.

The host, environment, and time can influence the microbiome composition [26]. However, in general, the host is the most significant factor influencing the microbial community. Despite holding a divergent abundance of a few genera, there were no significant differences in the microbial community over the depth range evaluated for *T. tagusensis*. The lack of variability might indicate that the sampled bathymetric gradient (i.e. differences in the environmental variable depth here) was not enough to significantly alter the microbiota associated to the corals, or that the microorganism community associated to *T. tagusensis* is shaped by the host and does not respond to small scale environmental factors, as noted for other species [26]. Although similar results have been documented for *Agaricia grahamae* sampled from different depths (55 m and 85 m) [25], a bathymetric gradient influenced the composition of the microbiome of *Madracis pharensis* and *Stephanocoenia intersepta* [25]. Another factor that might influence our results is the fact that the invasive population of *T. tagusensis* is highly clonal [55] and, therefore, it is possible that all analyzed specimens were genetically identical. Indeed, when comparing the microbiome of adult colonies to that from larvae, we found that they host a similar microbial community and share part of the microbial core, as proposed in the hologenome theory [27], thus supporting the idea that part of the microbiome is vertically transmitted as shown to occur in *Porites astreoides* [28] and *Acropora digitifera* [21]*.*

Therefore, invasive populations of *T. tagusensis* have two types of microbiome acquisition: the vertical, based on the transmission of the microbial core from the mother colonies to the larvae; and the horizontal transmission supported by the similarity between the microbial profile found in the region’s seawater and part of the colonies’ microbiome. Such mixed acquisition is similar to that observed in *Acropora digitifera* [21]. Vertical transmission indicates that the relationship between the core microbiome and the host is somewhat guaranteed over generations [21] and that the maintenance of beneficial symbionts [56] influence co-evolution. On the other hand, although horizontal acquisition can also be seen as evolution, it has a much shorter response time as it fluctuates following environmental factors. Thus, if the host acquires the symbionts exclusively through generations, microbiome adaptations following environmental changes would not be observed resulting in potential “unsatisfactory” microbial community that would not improve fitness [56]. In this context, it is possible that the successful invasion of *T. tagusensis* in the Southwestern Atlantic*,* in addition to the aforementioned biological factors, includes the mixed transmission capacity of the microbiome, as a factor of rapid adaptation to the adverse conditions of the different environments that it has been settling.

## Conclusions

In summary, we present here a description of the symbiont community that lives associated with the invasive coral in Brazil, *Tubastraea tagusensis*. Genetic analyzes allowed us to identify that a small depth gradient is not an influential factor in altering the composition of the microbiota. We could observe a similarity between the profile of microorganisms found in the seawater and that of the colonies’ microbiome. In addition to identifying a persistent microbial core composed of eight genera for the adult specimens, it is shown that it is partially present in larvae. Therefore, these results indicate that *T. tagusensis* has vertical and horizontal transmission/acquisition and that this factor can be one of the conditions for its success as an invasive species.

## Materials and methods

### Sampling, PCR, and sequencing

Thirty-two colonies of *T. tagusensis* were sampled along a depth gradient (3, 6, and 8 m) in a vertical rocky shore at Ilha dos Búzios, the northern coast of São Paulo on October 9th, 2017 (20 colonies), kept in the freezer until extraction, and November 30th, 2017 (12 colonies), extracted as soon as they arrived at the laboratory. To evaluate the vertical transmission of microbiota, six colonies were kept separately in open-water aquarium systems until the release of larvae, which occurred 19 days after the colonies were placed in aquariums, with the aid of manual pipette. Total genomic DNA was extracted from the outer region of the calyx (containing soft tissue and skeleton) from all 32 samples and from a pool of 10 larvae released from a single colony, using the DNeasy Blood & Tissue kit (Qiagen) following the manufacturer’s instructions. The quality and concentration of DNA were verified by electrophoresis in agarose gel (1.5%) and spectrophotometer (NanoDrop 2000), respectively. 16S rRNA gene was amplified by polymerase chain reaction (PCR) using the universal primers 27F and 519R [57, 58]. Reaction contained 16 ng of DNA, 2.5 μl of 10X Advantage-2 PCR Buffer, 2.5 mM of dNTP Mix, 5 μM of each primer, 0.5 μl of Advantage-HF 2 polymerase, and ultrapure water up to a final volume of 25 μl. Reactions were performed according to the following cycling condition: initial denaturation step at 94 °C for 60s, followed by 30 cycles at 94 °C for 30s, 56 °C for 40s and 68 °C for 33 s, with a final extension at 68 °C for 33 s.

PCR products were purified using magnetic beads (Agencourt AMPure XP) following the manufacturer’s instructions and eluted on 50 μl of TE (10 mM Tris-HCl, 1 mM EDTA, pH 8.0). The concentration of purified amplicons was determined using Qubit® dsDNA BR Assay Kit. Libraries were prepared using the NEBNext Ultra II FS DNA Library Prep Kit and kept frozen until sequencing. Upon sequencing each library was quantified using Qubit® dsDNA HS Assay Kit and size verified using capillary electrophoresis (Bioanalyzer High Sensitivity DNA chip). Sequencing was performed using the MiSeq Nano kit v2 (500 cycles) at the Facility Center for Research from the University of São Paulo (CEFAP-USP). Sequences were deposited in the SRA database (PRJNA637639).

### Bioinformatics

Low-quality and short sequences (< 50pb) were removed using the SolexaQA++ [59]. Identical sequences were grouped using the Swarm software with d = 1 [60] and then classified using the classify.seqs command on the Mothur platform, with a bootstrap cutoff of 80 [61] using the database 16S Silva-v1.32 (DBS) [62]. Statistical analyses were performed using the Statistical Analysis of Metagenomic Profiles software (STAMP - Parks et al. [63]) after removing eukaryote, chloroplast, mitochondria, and unknown sequences. Statistical analyses were performed through variance analyses (ANOVA) and post-hoc Turkey-Kramer tests, with Benjamini-Hochberg FDR correction of multiple tests, and *p*-value < 0.01 to identify the relative frequency of OTUs by the depth and significant differences between groups (3, 6, and 8 m). Whites’ two-sided nonparametric t-test, confidence interval method (DP bootstrap, Benjamini-Hochberg FDR multiple test correction and p-value filter of 0.01) were applied for comparisons between the groups of the adult colonies and larvae, and also between depths (i.e., 3 - 6 m, 3 - 8 m, and 6 - 8 m). Diversity parameters were calculated from the classification tables (phylum, class, order, family, and gender) such as richness estimators Shannon, Neff Shannon, Simpson, and Neff Simpson (inverse Simpson index).

## Supplementary information

**Additional file 1: Table S1.** Table with identification taxonomic of the microbiome associated with colonies, including genus, family, order, class, phylum, domain and sequences for each sample.

**Additional file 2: Figure S1.** Microbiome associated with the invasive colonies of *Tubastraea tagusensis*. Interactive Figure [44].

## Data Availability

All data generated or analysed during this study are included in this published article [and its supplementary information files].
