# Supplementary material for: Microbiome of the Southwestern Atlantic invasive scleractinian coral, Tubastraea tagusensis
Source: Anim Microbiome. 2020 Aug 11;2:29. doi: 10.1186/s42523-020-00047-3 (PMC7807860; doi:10.1186/s42523-020-00047-3)
Supplement: Supplementary file 2 — Additional file 2: Figure S1. Microbiome associated with the invasive colonies of Tubastraea tagusensis. Interactive Figure [44]. [file 42523_2020_47_MOESM2_ESM.html]

Javascript must be enabled to view this page.

magnitude

 100.000000000047

 100.000000000047

 46.1292294660321

 46.0929105395918

 44.1844792026

 44.1844792026

 44.1844792026

 1.3230182164582

 1.3230182164582

 .637992706485

 .614822597291

 .0702029126822

 .167734065264

 .167734065264

 .167734065264

 .35070918899796

 .132803691008

 .132803691008

 .1368805574548

 .0838863868136

 .0529941706412

 .05188164237113

 .0421407501054

 .00974089226573

 .0194024058983

 .0194024058983

 .00974089226573

 .00974089226573

 .0264970853206

 .0264970853206

 .0264970853206

 .0210703750527

 .0210703750527

 .0210703750527

 .0194024058983

 .0194024058983

 .0194024058983

 .0210703750527

 .0210703750527

 .0210703750527

 .0210703750527

 .0152485513876

 .0152485513876

 .0152485513876

 .0152485513876

 27.8612864987

 27.8612864987

 27.8612864987

 27.8612864987

 27.8612864987

 18.2122997093514

 11.4566957549788

 4.9770611159922

 4.8296152596386

 4.12573184882

 .688634859431

 .0152485513876

 .1474458563536

 .09754634768

 .0498995086736

 1.48149491421

 1.48149491421

 1.48149491421

 .680171491423

 .680171491423

 .581961801822

 .098209689601

 1.5678159154519

 .4574565416283

 .350716681915

 .0762427569381

 .0152485513876

 .0152485513876

 .326253988156

 .326253988156

 .310400843143

 .191136231102

 .10205587

 .017208742041

 .264224840553

 .137236962489

 .0812422239011

 .0457456541629

 .117988393646

 .117988393646

 .0457456541628

 .0304971027752

 .0152485513876

 .0304971027752

 .0304971027752

 .0152485513876

 .0152485513876

 .8356067034642

 .267063598815

 .267063598815

 .423670572937

 .138603571629

 .0776096235933

 .0458994912189

 .0458994912189

 .0391014737957

 .0308112673184

 .0304971027752

 .0152485513876

 .0529941706412

 .0529941706412

 .0529941706412

 .0529941706412

 .0194817845315

 .0194817845315

 .0194024058983

 .0194024058983

 .4358073004513

 .2881441607318

 .219901438703

 .0264970853206

 .0264970853206

 .0152485513876

 .0609942055505

 .0609942055505

 .0363189264403

 .0363189264403

 .0351014563411

 .0351014563411

 .0152485513876

 .0152485513876

 .3490894710555

 .3490894710555

 .201562665848

 .0767917073913

 .040237995041

 .0152485513876

 .0152485513876

 .4553773426751

 .3111864757763

 .172349430943

 .119434638935

 .0194024058983

 .0762427569381

 .0762427569381

 .0485457040624

 .0485457040624

 .0194024058983

 .0194024058983

 .158986611786

 .158986611786

 .158986611786

 .0543500251833

 .0543500251833

 .0543500251833

 .0682427220288

 .0682427220288

 .0529941706412

 .0152485513876

 .14307603118288

 .1344716601624

 .0507451211258

 .0459788698521

 .0194024058983

 .0183452632862

 .00860437102048

 .00860437102048

 .0388048117967

 .0388048117967

 .0388048117967

 .0388048117967

 .0388048117967

 .0388048117967

 .0518816423711

 .0518816423711

 .0308112673184

 .0210703750527

 .0296747460732

 .0296747460732

 .0296747460732

 .0264970853206

 .0264970853206

 .0264970853206

 .038963569063

 .038963569063

 .0194817845315

 .0194817845315

 .0152485513876

 .0152485513876

 .0152485513876

 .00974089226573

 .00974089226573

 .00974089226573

 5.86706742126403

 2.16989071609428

 2.16989071609428

 1.82594379446

 .254792102109

 .0458994912189

 .0194024058983

 .0152485513876

 .00860437102048

 .895079947789

 .895079947789

 .895079947789

 .35834271603766

 .34973834501718

 .279535432335

 .0615985416617

 .00860437102048

 .00860437102048

 .00860437102048

 .5900336765853

 .5900336765853

 .201284819099

 .14503442942

 .121023229456

 .0421407501054

 .0388048117967

 .0264970853206

 .0152485513876

 .84739883317163

 .28597365685273

 .169339067818

 .106893696769

 .00974089226573

 .105988341282

 .105988341282

 .1501153249472

 .0771633075234

 .0729520174238

 .2149492204652

 .063211125158

 .0629543962038

 .0388048117967

 .0347303359191

 .0152485513876

 .040472780951

 .040472780951

 .0498995086735

 .0304971027752

 .0194024058983

 .2249262113467

 .163383055343

 .163383055343

 .0421407501054

 .0421407501054

 .0194024058983

 .0194024058983

 .14108979762348

 .132485426603

 .132485426603

 .00860437102048

 .00860437102048

 .2610586716233

 .2458101202357

 .126375481301

 .0929375536141

 .0264970853206

 .0152485513876

 .0152485513876

 .1527610580808

 .102015936955

 .102015936955

 .0507451211258

 .0507451211258

 .11641443539

 .11641443539

 .0776096235933

 .0388048117967

 .040472780951

 .040472780951

 .040472780951

 .0304971027752

 .0304971027752

 .0304971027752

 .0304971027752

 .0304971027752

 .0304971027752

 .00860437102048

 .00860437102048

 .00860437102048

 .873287981721

 .873287981721

 .873287981721

 .873287981721

 .0152485513876

 .0152485513876

 .0152485513876

 .0152485513876

 3.18583287643303

 3.03394504393213

 2.02470636990564

 1.82667729013426

 .89750321493

 .299155782543

 .102975107879

 .0822168474384

 .063211125158

 .058207217695

 .0569941880959

 .0515674778279

 .0498995086736

 .0391014737957

 .0264970853206

 .0238529224081

 .0194024058983

 .0194024058983

 .017208742041

 .00974089226573

 .00974089226573

 .0822184176592

 .0822184176592

 .0919577397041

 .0529941706412

 .0389635690629

 .02385292240808

 .0152485513876

 .00860437102048

 .57509263010318

 .5301693326424

 .469997483794

 .0601718488484

 .0363189264403

 .0363189264403

 .00860437102048

 .00860437102048

 .165849867935

 .165849867935

 .165849867935

 .21831728868161

 .17921581488593

 .138977819845

 .0152485513876

 .0152485513876

 .00974089226573

 .03910147379568

 .0152485513876

 .0152485513876

 .00860437102048

 .0499788873067

 .0499788873067

 .0304971027752

 .0194817845315

 .1324854266026

 .1324854266026

 .105988341282

 .105988341282

 .0264970853206

 .0264970853206

 .0194024058983

 .0194024058983

 .0194024058983

 .0194024058983

 2.07182702390716

 .684138060459

 .684138060459

 .684138060459

 .684138060459

 1.24512086690973

 .534043504922

 .534043504922

 .534043504922

 .4393577272237

 .2826796504734

 .171901064942

 .068637835426

 .0421407501054

 .0723965765395

 .0529941706412

 .0194024058983

 .0421407501054

 .0421407501054

 .0210703750527

 .0210703750527

 .0210703750527

 .0210703750527

 .16300413178423

 .13650704646363

 .0588180442372

 .0485457040624

 .0194024058983

 .00974089226573

 .0264970853206

 .0264970853206

 .0529941706412

 .0529941706412

 .0529941706412

 .040472780951

 .040472780951

 .0210703750527

 .0194024058983

 .0152485513876

 .0152485513876

 .0152485513876

 .11175682922

 .11175682922

 .11175682922

 .11175682922

 .0210703750527

 .0210703750527

 .0210703750527

 .0210703750527

 .00974089226573

 .00974089226573

 .00974089226573

 .00974089226573

 1.16276577410009

 1.10227976070849

 .54441703350776

 .350522735482

 .350522735482

 .0723965765396

 .0723965765396

 .07295201742373

 .063211125158

 .00974089226573

 .0388048117967

 .0388048117967

 .00974089226573

 .00974089226573

 .52871942903663

 .3584057250337

 .337335349981

 .0210703750527

 .106752921757

 .106752921757

 .0538198899802

 .0344174840819

 .0194024058983

 .00974089226573

 .00974089226573

 .0291432981641

 .0291432981641

 .0291432981641

 .0421407501054

 .0421407501054

 .0421407501054

 .0421407501054

 .0183452632862

 .0183452632862

 .0183452632862

 .0183452632862

 .6317517001665

 .4648289794917

 .3924324029522

 .3924324029522

 .227462069794

 .135295587085

 .0296747460732

 .0529941706412

 .0529941706412

 .0529941706412

 .0194024058983

 .0194024058983

 .0194024058983

 .0529941706412

 .0529941706412

 .0529941706412

 .0529941706412

 .0734557690826

 .0734557690826

 .0734557690826

 .0388048117967

 .0194024058983

 .0152485513876

 .0210703750527

 .0210703750527

 .0210703750527

 .0210703750527

 .0194024058983

 .0194024058983

 .0194024058983

 .0194024058983

 .1594052384476

 .1594052384476

 .1594052384476

 .14415668706

 .14415668706

 .0152485513876

 .0152485513876

 .10237003454268

 .08129965948998

 .0726952884695

 .0726952884695

 .0726952884695

 .00860437102048

 .00860437102048

 .00860437102048

 .0210703750527

 .0210703750527

 .0210703750527

 .0210703750527

 .0723965765396

 .0723965765396

 .0723965765396

 .0723965765396

 .0723965765396

 .25635439220928

 .25635439220928

 .1642587477724

 .069518940423

 .069518940423

 .0682427220288

 .0682427220288

 .0264970853206

 .0264970853206

 .06559855911628

 .0569941880958

 .0417456367082

 .0152485513876

 .00860437102048

 .00860437102048

 .0264970853206

 .0264970853206

 .0264970853206

 .07687108602443

 .07687108602443

 .07687108602443

 .0671301937587

 .0518816423711

 .0152485513876

 .00974089226573

 .00974089226573

 .0388048117967

 .0388048117967

 .0388048117967

 .0388048117967

 .0388048117967

 .0194024058983

 .0194024058983

 .0194024058983

 .0194024058983

 .0194024058983

 .0194024058983

 .0194024058983

 .0194024058983

 .0194024058983

 .0194024058983
